# Intramolecular multi-bond strain: the unrecognized side of the dichotomy of conjugated systems

**DOI:** 10.1039/c6sc00454g

**Published:** 2016-05-20

**Authors:** Yirong Mo, Huaiyu Zhang, Peifeng Su, Peter D. Jarowski, Wei Wu

**Affiliations:** a The State Key Laboratory of Physical Chemistry of Solid Surfaces , Fujian Provincial Key Laboratory of Theoretical and Computational Chemistry and College of Chemistry and Chemical Engineering , Xiamen University , Xiamen , Fujian 361005 , China; b Department of Chemistry , Western Michigan University , Kalamazoo , Michigan 49008 , USA . Email: ymo@wmich.edu; c University of Surrey , Advanced Technology Institute , Guildford , GU2 7XH , UK . Email: peterjarowski@gmail.com; d iChEM , Xiamen University , Xiamen , Fujian 361005 , China . Email: weiwu@xmu.edu.cn

## Abstract

Electron conjugation stabilizes unsaturated systems and diminishes the differences among bond distances. Experimentally, Kistiakowsky and coworkers first measured and noticed the difference between the hydrogenation heats of carbon–carbon double bonds in conjugated systems. For instance, the hydrogenation heat of butadiene is 57.1 kcal mol^–1^, which is less than two times that of the hydrogenation heat of 1-butene (30.3 kcal mol^–1^), and the difference (3.5 kcal mol^–1^) is the extra stabilization due to the resonance between two double bonds in the former, and is referred to as the experimental resonance energy. Following Kistiakowsky's definition, Rogers *et al.* studied the stepwise hydrogenation of 1,3-butadiyne and concluded that there is no conjugation stabilization in this molecule. This claim received objections instantly, but Rogers and coworkers further showed the destabilizing conjugation in 2,3-butanedione and cyanogen. Within resonance theory, the conjugation energy is derived “by subtracting the actual energy of the molecule in question from that of the most stable contributing structure.” The notable difference between the experimental and theoretical resonance energies lies in that the former needs other real reference molecules while the latter does not. Here we propose and validate a new concept, intramolecular multi-bond strain, which refers to the repulsion among π bonds. The π–π repulsion, which is contributed to by both Pauli exchange and electrostatic interaction, is quantified with the B_4_H_2_ model system (16.9 kcal mol^–1^), and is compared with the σ–σ repulsion in B_2_H_4_ (7.7 kcal mol^–1^). The significance of the π–π repulsion can be demonstrated by the much longer carbon–nitrogen bond in nitrobenzene (1.486 Å) than in aniline (1.407 Å), the very long and weak nitrogen–nitrogen bond (1.756 Å) in dinitrogen tetroxide, and the instability of long polyynes. This new concept successfully reconciles the discrepancy between experimental and theoretical conjugation energies. However, we maintain that by definition, electron conjugation must be stabilizing.

## Abstract 2

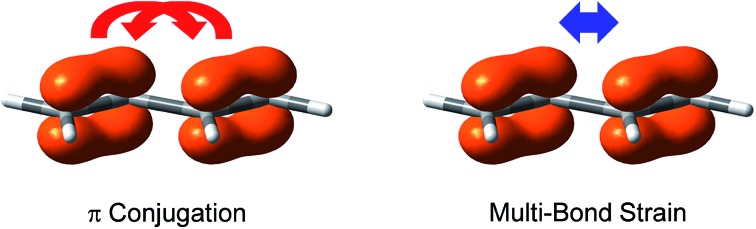
Apart from the more familiar π-conjugation, there is also significant π–π repulsion which is a kind of unrecognized intramolecular strain and can be quantified with the linear B_4_H_2_ model system.

## 1. Introduction

Ever since the proposal of strain theory by Baeyer in 1885,^1^ the concept of molecular strain has been well accepted as one of the key factors influencing the preference of molecular conformations and reactivity.^2^–^10^ Strain energy is often derived as the difference between the experimental heat of formation for a strained molecule and the expected heat of formation for a hypothetical strain-free molecule with the same number of atoms which is derived from group contributions of additivity methods^11^,^12^ or *ab initio* computations.^13^,^14^ Strain is also addressed in terms of bond length, bond angle, torsional angle and noncovalent interactions. In general, molecular strain can be classified as either ring strain, such as in cyclopropane,^14^–^19^ or steric strain, such as in eclipsed ethane.^20^–^25^

Different from destabilizing molecular strains, electron delocalization refers to the electron transfer from one moiety to another, and includes hyperconjugation and conjugation based on the symmetry of the orbitals involved. A notable example in this regard is conjugated linear molecules.^26^ By definition, electron delocalization must be a stabilizing factor, otherwise nature would choose an electron localized structure. This effect has been well recognized in conjugated systems such as graphene and conductive polymers and many organic pigments which are widely used in organo-luminescent devices and dye-sensitized solar cells. A conjugated system cannot be well described with a single Lewis structure where each bonding pair of electrons is localized on no more than two atoms and thus a resonance theory is needed.^27^,^28^ Theoretically, conjugation (resonance) energy can be derived “by subtracting the actual energy of the molecule in question from that of the most stable contributing structure”^28^ using *ab initio* valence bond (VB) theory.^29^–^32^ The popular molecular orbital (MO) theory, however, has difficulties in defining electron-localized states for references in the way VB theory does,^33^ though approximate approaches have been developed particularly in the study of electron transfer processes.^34^–^38^ Alternatively, other reference molecules have to be chosen and various isodesmic and homodesmotic model reactions need to be designed to estimate resonance energies,^39^ but the intrusion of other effects in the reference systems, such as strain, hyperconjugation, Coulomb repulsion imbalance, and uncompensated van der Waals (dispersion) attractions, has been recognized.^40^,^41^ Experimentally, Kistiakowsky and coworkers first measured and noticed the differences between the hydrogenation heats of the carbon–carbon double bonds in substituted and/or conjugated systems.^42^,^43^ For instance, the hydrogenation heat of butadiene is 57.1 kcal mol^–1^, which is less than two times the hydrogenation heat of 1-butene (30.3 kcal mol^–1^), and the difference (3.5 kcal mol^–1^) is the extra stabilization due to the resonance between two double bonds in the former, and is referred to as the Kistiakowsky resonance energy. The process can be expressed as the sequential hydrogenation in the following steps (in kcal mol^–1^)^43^
1

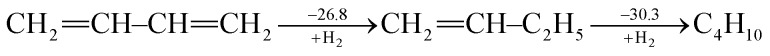




If we take ethylene instead of 1-butene as the reference, however, the difference would be 8.5 kcal mol^–1^.

Following Kistiakowsky's definition,^42^ Rogers *et al.* computationally studied the stepwise hydrogenation of 1,3-butadiyne as
2

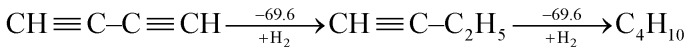

and concluded that there is no conjugation stabilization in this molecule.^44^,^45^ Even if we use acetylene as a reference, the difference is 10.0 kcal mol^–1^, which is close to, rather than twice, the value for butadiene with reference to ethylene (8.5 kcal mol^–1^). Based on resonance theory, we would expect that the thermodynamic conjugation stabilization in 1,3-butadiyne with two Π44 (4-electron-4-center) bonds to be two times that of the quantity in 1,3-butadiene with only one Π44 bond, as demonstrated first by Kollmar who derived the resonance stabilization energies in 1,3-butadiene and 1,3-butadiyne as 9.7 and 19.1 kcal mol^–1^, respectively, by replacing their π MOs with the π MOs of ethylene and ethyne.^46^

Rogers' claim that the conjugation stabilization in 1,3-butadiyne is zero received objections instantly.^47^–^49^ Jarowski *et al.* pointed out that there is significant hyperconjugation from the ethyl group to the triple bond (*i.e.*, σ → π*) in 1-butyne.^48^ This stabilizing force leads to an underestimation of the conjugation with Kistiakowsky's definition. Jarowski *et al.* predicted conjugation energies of 9.3 kcal mol^–1^ for diynes and 8.2 kcal mol^–1^ for dienes. Based on the energy decomposition analysis between two fragments (˙C≡CH and ˙CH=CH_2_), Cappel *et al.* estimated the conjugative stabilization in 1,3-butadiyne (45.0 kcal mol^–1^) to be about twice the value of that in 1,3-butadiene (19.5 kcal mol^–1^).^47^ Nevertheless, Rogers *et al.* continued their work and showed the cases of 2,3-butanedione and cyanogen where the conjugation is even destabilizing, *e.g.*
3

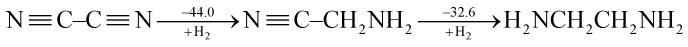




Rogers surmised that the lack of overall thermodynamic stabilization in polyynes is due to the repulsions among the six electrons of each triple bond.^50^ But this kind of interaction exists in ethyne as well and the extra stabilization in 1,3-butadiyne with reference to ethyne is still only 10.0 kcal mol^–1^.

A molecular structure results from a balance of repulsive and attractive forces. Electron delocalization is an electronic effect and concerns charge transfer from an occupied bond orbital to vicinal unoccupied anti-bond orbitals, while a steric effect reflects the interaction between neighbouring occupied bond orbitals, and generally comprises the classical electrostatic (*e.g.*, local dipole–dipole interaction) term and quantum mechanical Pauli exchange repulsion. Thus, by definition, conjugation must be stabilizing. The seeming lack of stabilization or even destabilization found by Rogers *et al.* must result from a certain unrecognized repulsion. Here we propose and demonstrate a new concept, intramolecular multi-bond strain, as the source of significant repulsion among π bonds, which has not been well appreciated.

## 2. Synopsis

**Fig. 1:**
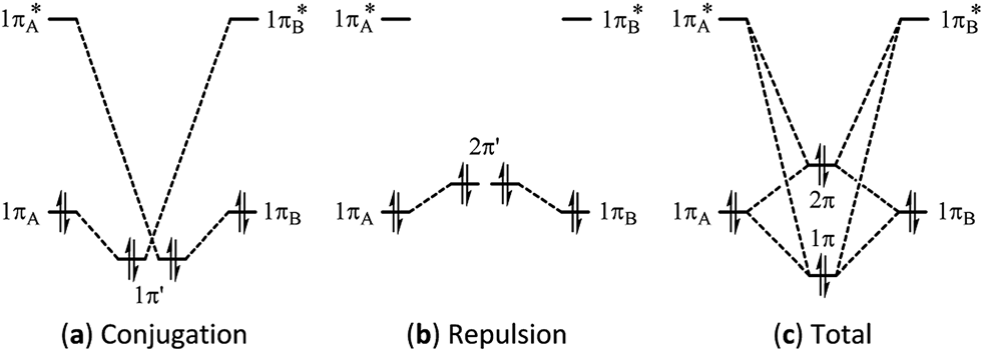
Orbital interaction diagrams showing (a) conjugation, (b) repulsion and (c) the sum of conjugation and repulsion in two symmetrical conjugated π bonds.

Multi-bond strain refers to the repulsion among conjugated π bonds or hyperconjugated σ–π bonds that, up until now, has not been identified with only the stabilizing conjugative or hyperconjugative interactions being generally recognized. The coexistence of the stabilizing and destabilizing forces can be better described by the following orbital interaction diagrams (Fig. 1). We consider two neighbouring π bonds on fragments A and B. The conjugation occurs between an occupied orbital (π_A_ or π_B_) and the other moiety's virtual orbital (π*B or π*A) as shown in Fig. 1a. This has been the focal point for π conjugation, and *ab initio* valence bond (VB) methods,^29^–^32^,^51^ including our block-localized wavefunction (BLW)^52^–^54^ can evaluate the magnitude of this stabilizing effect. However, the interaction between occupied orbitals π_A_ and π_B_ cannot be neglected; this interaction is obviously repulsive and is usually termed steric repulsion (Fig. 1b). We emphasize that the steric effect is generally composed of both Pauli exchange repulsion and local dipole–dipole electrostatic interactions, and multi-bond strain exactly reflects this destabilizing factor. The overall π–π interaction results from both the stabilizing and destabilizing components, as shown in Fig. 1c. The relative stability of 1π *versus* the destability of 2π reflects Kistiakowsky's experimental resonance energy, whereas Fig. 1a highlights the Pauling–Wheland definition of theoretical resonance energy. Thus, the discrepancy between the experimental and theoretical definitions of resonance is the repulsion shown in Fig. 1b.

## 3. Estimation of the repulsive interaction

**Fig. 2:**
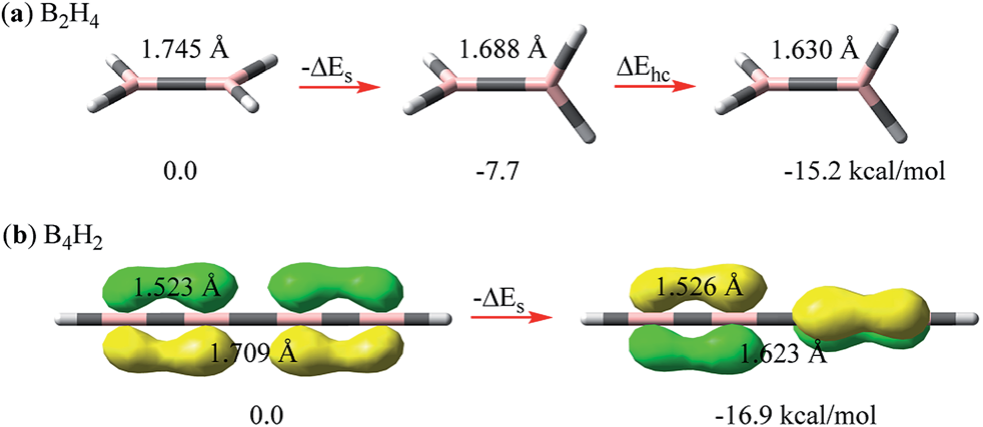
Energetic and structural changes along the rotation of interacting moieties (either σ or π bonds) from a parallel to perpendicular orientation for (a) B_2_H_4_ and (b) B_4_H_2_.

Here we compare the relative σ–σ and π–π repulsions with model systems of linear B_2_H_4_ (ref. 55–57) and B_4_H_2_ at the B3LYP/6-311+G(d,p) level using the BLW method^52^–^54^ which is the simplest variant of *ab initio* valence bond (VB) theory. The reason that we chose boron systems here is that the boron atom is electron-deficient and thus we can strictly keep its certain p orbitals unoccupied and examine the evolution of repulsive interactions with little interference, while importantly, the neutrality of these systems is maintained, *e.g.*, in planar B_2_H_4_, there is no π electron in the p_π_ orbitals. By keeping these p_π_ orbitals vacant,^57^ we rotate one BH_2_ group to the perpendicular conformation. The energy lowering in this process can be solely ascribed to the relief of the σ–σ repulsion between BH bonds (Δ*E*_s_ in Fig. 2a). Computations show that the energy reduction is 7.7 kcal mol^–1^, accompanied by a boron–boron bond shortening of 0.057 Å. This energetics is considerably higher than the rotation barrier in ethane (2.7 kcal mol^–1^ at the same theoretical level) as there is still significant repulsion even in the latter's staggered conformation. We note that Δ*E*_s_ is contributed to by both the Pauli repulsion and electrostatic repulsion as the BH bond is slightly polar. The introduction of the σ–π* hyperconjugative interaction would further shorten the BB bond and stabilize the perpendicular conformation by 7.5 kcal mol^–1^. In total the rotation barrier in B_2_H_4_ is 15.2 kcal mol^–1^.

Linear B_4_H_2_ (HB=B–B=BH) offers an interesting opportunity to directly probe the π–π repulsion with two localized π pairs that are either parallel or perpendicularly arranged. The ground state of linear B_4_H_2_ has two 4-center-2-electron (Π24) bonds, and if we strictly localize each pair on two terminal B–B bonds with the BLW method,^52^–^54^ we derive the localized state with two perpendicular π bonds (shown on the right side of Fig. 2b). The double bond and central single bond distances are 1.526 and 1.623 Å, respectively. Differently, we can also arrange the two π bonds in the same direction. This results in an enhanced repulsion between them and the strictly localized system is destabilized by 16.9 kcal mol^–1^. The comparison of B_2_H_4_ and B_4_H_2_ reveals that the π–π repulsion is possibly stronger than the σ–σ repulsion. Accordingly, the central B–B single bond stretches by 0.086 Å to 1.709 Å. This surprisingly high repulsion signifies the strain in all conjugated systems. The delocalization between the π bonds, much like in butadiene, stabilizes the system by 10.8 kcal mol^–1^ and shortens the central bond by 0.066 Å. Notably, since the π–π resonance stabilization in the Π44 state of B_4_H_2_ is less than the π–π repulsion, this molecule would exhibit destabilizing conjugation following eqn (3).

It should also be pointed out that the π–π repulsion has been long implicated in the discussion of π-distortivity in benzene,^58^–^63^ the seminal example of aromaticity, notably by Shaik and Hiberty,^64^–^67^ who concluded that the π-electronic component of benzene prefers a localizing distortion with alternating bond lengths and the symmetrical structure with equal bonds is actually imposed by the σ electrons, though the π-electronic resonance necessarily stabilizes the system. Their concept of the dual character of conjugated systems is generally reflected in Fig. 1. In other words, the nature of the distortive tendency of the π-component in benzene results from the π–π repulsion among the three π bonds in the Kekulé structure.

## 4. Indicators of the π–π repulsion

**Fig. 3:**
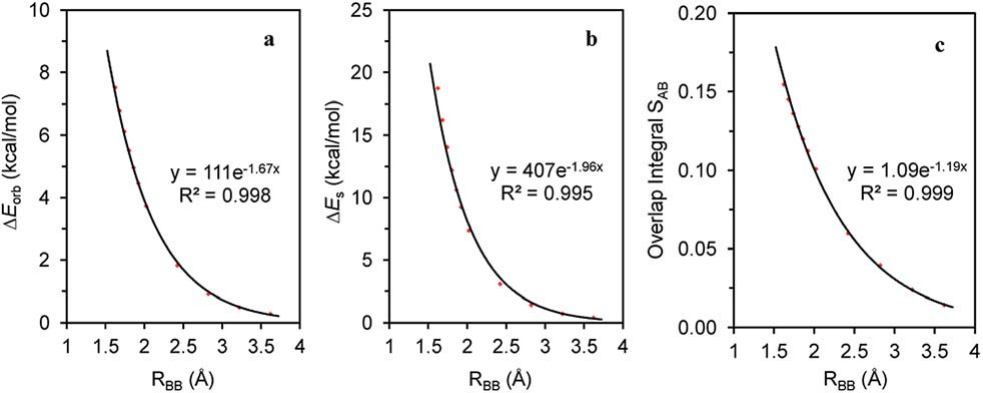
Correlations of (a) Δ*E*_orb_; (b) Δ*E*_s_ and (c) overlap integral *S*_12_ with the central BB bond distance in the B_4_H_2_ model system.

The magnitude of the stabilizing conjugation effect can be evaluated with the reference of the most stable resonance structure which can be well defined using VB theory. The estimation of the intramolecular repulsion, however, seems challenging as there is no obvious reference. This is unlike the study of intermolecular interactions where we take the separated monomers as the reference state, but at least for the model molecule B_4_H_2_, we can retain the molecular structure but change its electronic structure, and derive the localized orbital energies. When the two localized orbitals are perpendicular to each other (shown on the right side of Fig. 2b), we assign their orbital energies to 1π_A_ (or 1π_B_), as shown in Fig. 1. When the two localized orbitals are parallel to each other, their energies will be pushed up as 2π′ due to repulsion, as shown in the middle of Fig. 1. The energy difference Δ*E*_orb_ between 1π_A_ and 2π′ thus measures the strength of repulsion, and so is the overall energy difference Δ*E*_s_ between the two electronic states. Fig. 3 shows the correlations of Δ*E*_orb_ and Δ*E*_s_ with the variation of the central BB bond distance.

Fig. 3 shows the excellent exponential correlation of the energy terms with the bond distance. Considering that the energy terms are implicated by both the Pauli exchange and electrostatic interaction and the consensus that the Pauli repulsion increases exponentially as atomic wavefunctions decay exponentially,^68^ we speculate that in B_4_H_2_ the π–π repulsion is largely contributed to by the Pauli exchange repulsion. In addition, Fig. 3 indicates a linear relationship between the orbital energy difference (Δ*E*_orb_) and the steric energy (Δ*E*_s_). As expected, the overlap integral between the two π orbitals in the parallel orientation (1π_A_ and 1π_B_ in Fig. 1) is a good indicator of the steric repulsion in this case as a similar exponential correlation with the distance can be found (Fig. 3c).

## 5. Conjugation and repulsion in butadiene (**1**), butadiyne (**2**), cyanogen (**3**) and α-dicarbonyl (**4**)

**Fig. 4:**
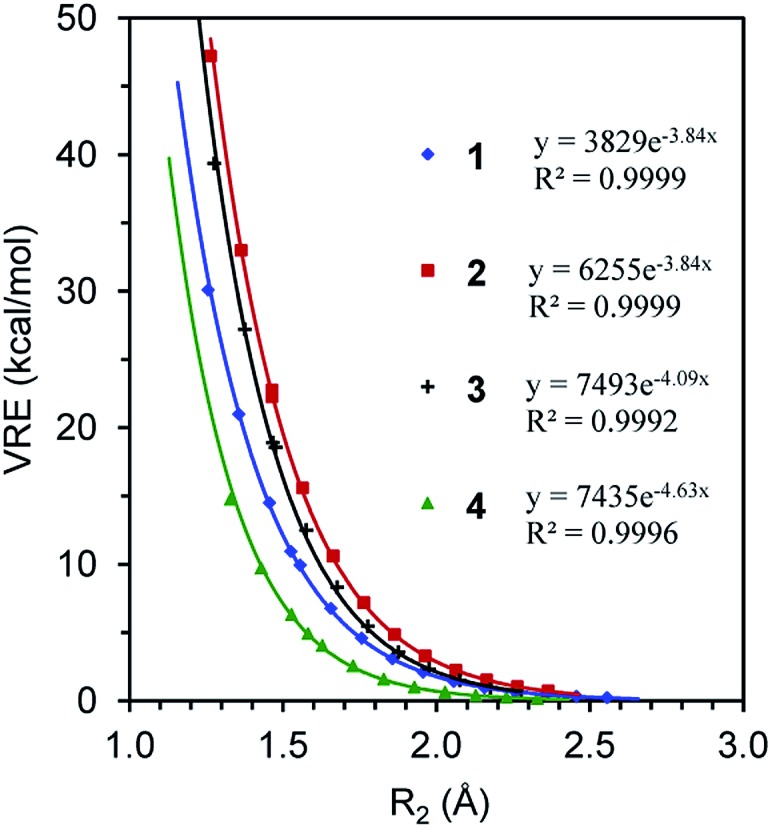
The exponential correlation of vertical resonance energy (VRE) with the central CC bond distance (*R*_2_) in butadiene (**1**), butadiyne (**2**), cyanogen (**3**) and α-dicarbonyl (**4**).

We first compare the resonance in the two most typical conjugated systems, butadiene and butadiyne. A comparison of the DFT and BLW optimizations shows that the localization of π electrons on their respective multiple bonds considerably stretches the central CC bonds by 0.071 and 0.101 Å, respectively for **1** and **2**, and the optimal distances reflect the intrinsically shorter Csp–Csp (1.465 Å) *versus* Csp^2^–Csp^2^ (1.528 Å) single σ bonds. In the meantime, the deactivation of resonance modestly shortens the double and triple bond lengths which are essentially the same as the bond distances in ethylene (1.329 Å) and acetylene (1.199 Å) at the same B3LYP/6-311+G(d,p) level. Theoretically, there are two types of resonance energies, namely vertical resonance energy (VRE) and adiabatic resonance energy (ARE). The former is the energy difference between DFT and BLW computations of the same structure, while the latter is the energy difference between the optimal delocalized state (*i.e.*, DFT optimization) and the optimal localized state (*i.e.*, BLW optimization). Fig. 4 shows the change in VRE along the central CC bond distance in the conjugated systems studied in this work. Much like in Fig. 3, there is an excellent exponential correlation between VRE and the central bond distance in each molecule.

In the DFT optimized geometries, the VRE of butadiyne (32.9 kcal mol^–1^) is a little more than two times that of the value in butadiene (14.5 kcal mol^–1^). This is in agreement with the studies by Kollmar,^46^ and Cappel *et al.*,^47^ and consistent with the facts that the central bond in butadiyne is shorter than in butadiene and there are two Π44 bonds in the former but only one in the latter. The ARE is supposed to be comparable to experimental resonance energies with reference to individual multiple bonds such as ethylene and acetylene. However, isodesmic reactions show that the experimental resonance energies (EREs) for butadiene (8.5 kcal mol^–1^) and butadiyne (10.0 kcal mol^–1^) are not only similar but also considerably lower than the theoretical AREs (12.6 and 27.0 kcal mol^–1^ for **1** and **2**, respectively). If we take the difference between ARE and ERE as the steric contribution, the steric repulsion in butadiene and butadiyne are 4.1 and 18.8 kcal mol^–1^ respectively. The latter is much more than two times the former, due to the much shortened central CC bond distance, and the repulsive force increases exponentially along the distance (Fig. 3). It should be noted that our AREs are very close to the evaluations of conjugation stabilization (14.8 and 27.1 kcal mol^–1^) in butadiene and butadiyne by Wodrich *et al.* who reinterpreted the differences in the hydrogenations of the first and second multi-bond in eqn (1) and (2) after introducing the “protobranching” concept.^69^

One way to estimate the intramolecular steric repulsion Δ*E*_s_ is the compression energy,^28^ which is the difference between the VRE of the optimal DFT structure and the ARE and reflects the energy cost for the structural change (dominated by the central CC single bond variation Δ*R*_2_) when conjugation is deactivated. Here we propose a force constant *k* to evaluate and compare the magnitude of the intramolecular steric repulsion
4

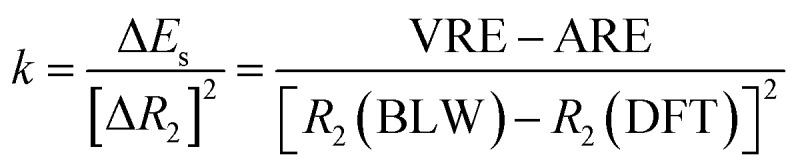




Table 1 shows that butadiyne has a much higher *k* value than butadiene. Of course, *k* measures the change of repulsion with the distance, *i.e.*, the repulsive force, rather than the absolute energetic value of the steric repulsion. While the π–π repulsion considerably offsets the theoretical resonance energy (ARE) and leads to the experimental resonance energy (ERE), the repulsion in butadiyne is much stronger than in butadiene. In the end, both molecules exhibit comparable EREs, and interestingly, as found by Rogers *et al.*,^44^,^45^ there is zero thermodynamic conjugation stabilization in butadiyne.

**Table 1:** Major optimal bond distances (Å) in delocalized (DFT) and localized (BLW) states and the computed resonance energies (VRE and ARE, in kcal mol^–1^) compared with experimental resonance energies (ERE, in kcal mol^–1^) and the force constant (kcal Å^–2^)

Molecule	State	*R*_1_	*R*_2_	VRE	ARE	ERE	*k*
Butadiene	DFT	1.338	1.457	14.5	12.6	8.5	382
	BLW	1.326	1.528	10.9			
Butadiyne	DFT	1.207	1.364	32.9	27.0	10.0	589
	BLW	1.194	1.465	22.2			
Cyanogen	DFT	1.155	1.376	27.2	22.7	–4.3^*a*^	576
	BLW	1.145	1.467	18.9			
α-Dicarbonyl	DFT	1.203	1.529	6.3	5.6	4.3^*a*^	380
	BLW	1.196	1.583	4.9			

^*a*^Reaction enthalpy at 298 K.

We further look at the cases of cyanogen (**3**) and α-dicarbonyl (**4**) where Rogers *et al.* showed even thermodynamic destabilization.^70^ Indeed, we confirmed a reduced conjugation stabilization in both systems, compared with butadiyne and butadiene, respectively. The central CC bond in cyanogen is 0.012 Å longer than that in butadiyne. But when π electrons are localized, the optimal central bond lengths are quite similar (1.465 *versus* 1.467 Å) and correspond to the Csp–Csp single σ bond. This suggests a stronger repulsion between the triple bonds in cyanogen than in butadiyne, most likely due to the polarity of the CO π bonds which lead to dipole–dipole electrostatic repulsion. In fact, if we use the isodesmic reaction eqn (5) to measure the experimental resonance energy, we find that the ERE is even negative at the G3 theoretical level. This is consistent with eqn (3) where the hydrogenation of the first cyano group is more exothermic than the hydrogenation of the second cyano group. The stretched central C–C bond in α-dicarbonyl is an example where the π–π electrostatic repulsion plays a big role.
5

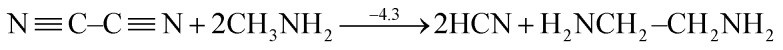



6

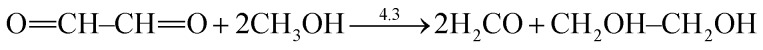




**Fig. 5:**
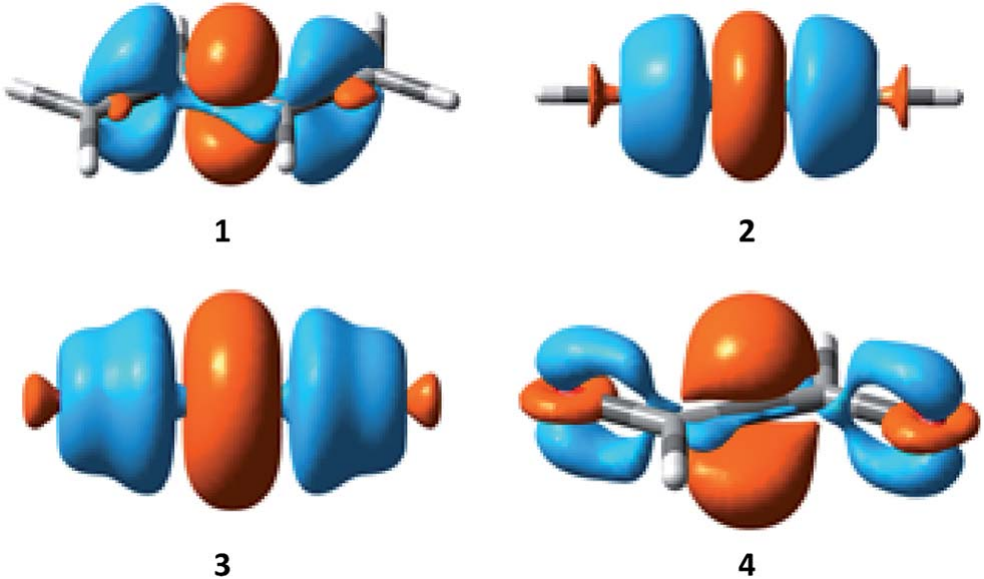
Electron density difference (EDD) maps showing the resonance in butadiene (**1**), butadiyne (**2**), cyanogen (**3**) and α-dicarbonyl (**4**).

Electron density difference (EDD) maps can be used to directly visualize the resonance, as the electron density difference between the electron-localized state (BLW) and the electron-delocalized state (DFT) reflects the movement of electron density. Fig. 5 plots the EDD maps of the four conjugated systems studied here. The orange color means a gain of electron density while the cyan color shows a loss of electron density. Following the conventional view, resonance moves the π electron density from multiple bonds to the linking single bonds, leading to the significant shortening of the central bonds.

## 6. Conclusion

Conjugation has been associated with high stability, planarity, small bond length alternation and many other physicochemical properties such as the bathochromic shift and lifetime of excited states. However, this is only one side of the story and concomitantly there is significant repulsion between π bonds which has not been well-recognized. In other words, while conjugation results in the stabilization of a conjugated system, the stability of the system is not solely determined by the conjugation, and there is intramolecular π–π repulsion which counteracts the stabilizing forces. This unrecognized repulsion is the culprit for the findings that conjugation has no stability or even is destabilizing. Considering that “strain, in a general sense, represents a cornerstone of the 150 year old field of conformational analysis”,^71^ here we propose the concept of intramolecular multi-bond strain to recognize the π–π repulsion. The magnitude of the π–π repulsion can be quantitatively assessed with the linear B_4_H_2_ (16.9 kcal mol^–1^), in comparison with the σ–σ repulsion in B_2_H_4_ (7.7 kcal mol^–1^). This new concept thus elucidates the difference between experimental and theoretical conjugation energies, although by definition, conjugation or resonance must be stabilizing.

Although quantum Pauli exchange repulsion is the primary cause for the π–π repulsion when the π bonds are nonpolar, local dipole–dipole repulsion can contribute and sometimes even dominate the π–π repulsion in cases with polarized π electron densities. The strong π–π repulsion is also implicated in numerous experimental findings. One notable example is the much longer carbon–nitrogen bond in nitrobenzene (1.486 Å) than in aniline (1.407 Å). Due to the resonance of the lone nitrogen pair to more electronegative oxygen atoms in the nitro group, there is a significant π dipole, which repels the π electron density in the benzene ring *via* both Pauli exchange and electrostatic interactions, leading to a long carbon–nitrogen bond in nitrobenzene.^72^ In fact, the strong π–π electrostatic repulsion, as shown by the much longer central C–C bond distance in α-dicarbonyl than in butadiene when conjugation is quenched (Table 1), is the major culprit for the remarkably stretched nitrogen–nitrogen bond in the weakly bound dinitrogen tetroxide (1.756 Å) compared with the single bond in hydrazine (1.47 Å).^73^ With both the electrostatic and Pauli repulsion deactivated, the nitrogen–nitrogen bond in dinitrogen tetroxide can be dramatically shortened to 1.471 Å as optimized by the BLW method at the B3LYP/6-311+G(d,p) level.^72^ The well-recognized instability of long polyynes should be contributed to by the strong intramolecular multi-bond strain as well.^74^–^77^

## 7. Methodology

The block-localized wavefunction (BLW) method originates from *ab initio* valence bond (VB) theory as it simplifies the original Heitler–London–Slater–Pauling (HLSP) function to a BLW of only one Slater determinant form with block-localized MOs. Orbitals in the same subspace are subject to the orthogonality constraint, but orbitals belonging to different subspaces are nonorthogonal. Thus, the BLW method combines the advantages or features of both MO and VB theories. In general, for an electron-localized state which is usually the most stable resonance structure, we partition the system to *k* blocks and define its wavefunction with a BLW (here we assume that the number of electrons in each block is even (equal to 2*n*_*i*_) and thus orbitals are doubly occupied) as

7 *Ψ*^BLW^ = det|*φ*_11_^2^*φ*_12_^2^…*φ*_1*n*_1__^2^…*φ*_*i*1_^2^…*φ*_*in*_*i*__^2^…*φ*_*kn*_*k*__^2^| = *Â*[*Φ*_1_…*Φ*_*i*_…*Φ*_*k*_]

where

8 *Φ*_*i*_ = *Â*[*φ*_*i*1_^2^*φ*_*i*2_^2^…*φ*_*in*_*i*__^2^]

is defined for the block *i*. For the cases studied in this work, apart from the σ frame which forms one block, each block is composed of two π electrons on two atoms. The resonance energy (RE) is defined as

9 RE = *E*(*Ψ*^BLW^) – *E*(*Ψ*^DFT^)

Geometry optimizations and calculations for adiabatic states with the regular DFT and diabatic states with the BLW method were performed with our in-house version of the quantum mechanical software GAMESS.^78^
